# Supplementary figures and images for: Differential expression of gut protein genes and population density of Arsenophonus contributes to sex-biased transmission of Bemisia tabaci vectored Cotton leaf curl virus
Source: PLoS One. 2021 Nov 29;16(11):e0259374. doi: 10.1371/journal.pone.0259374 (PMC8629229; doi:10.1371/journal.pone.0259374)

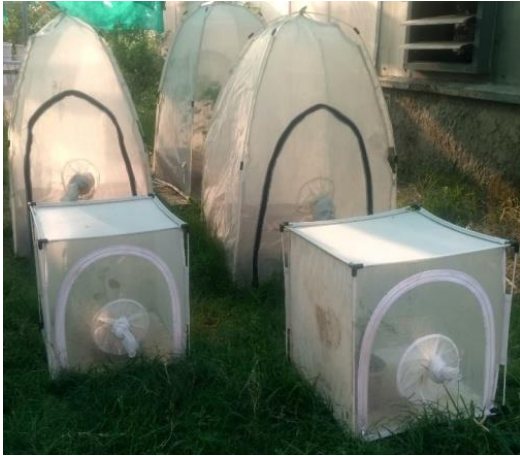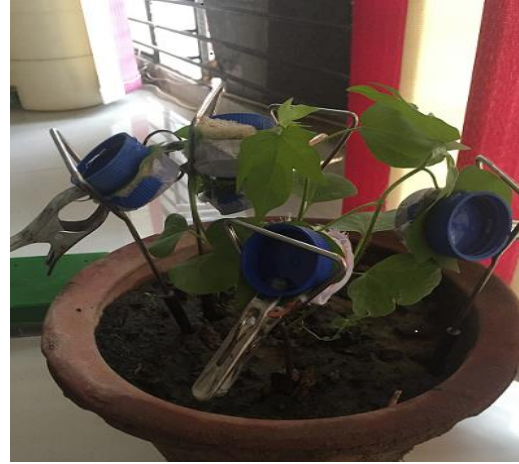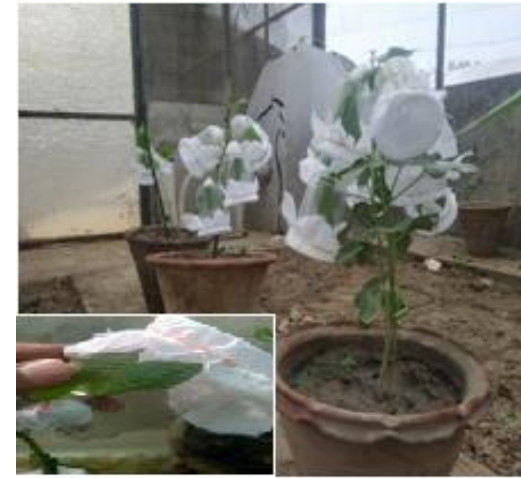

Supplement: S1 Fig — A. Maintenance of virus free plants inside insect proof cages B. Clip inoculation setup for whitefly inoculation C. Cup cages for whitefly inoculation. (PDF) [file pone.0259374.s001.pdf]

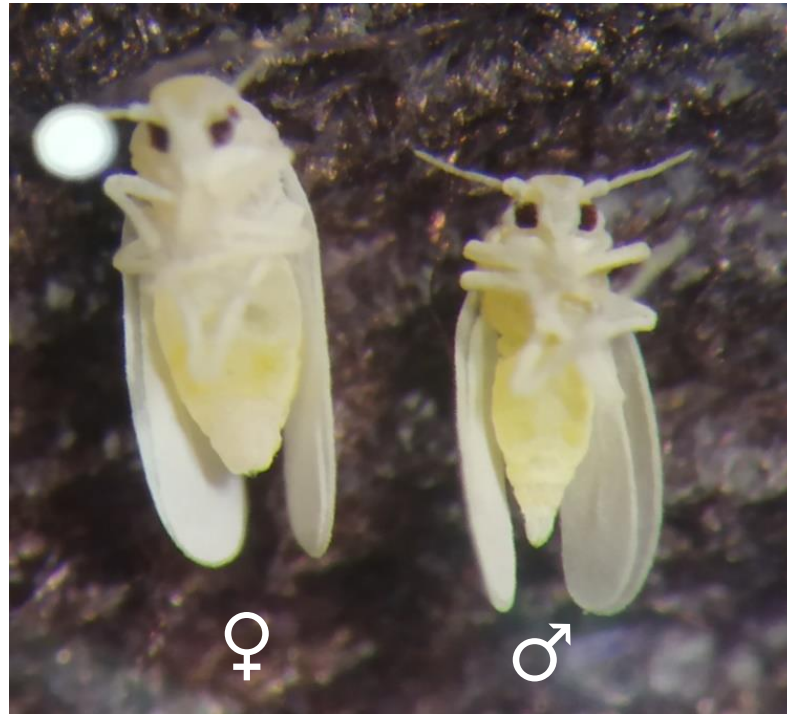

Supplement: S2 Fig — (PDF) [file pone.0259374.s002.pdf]

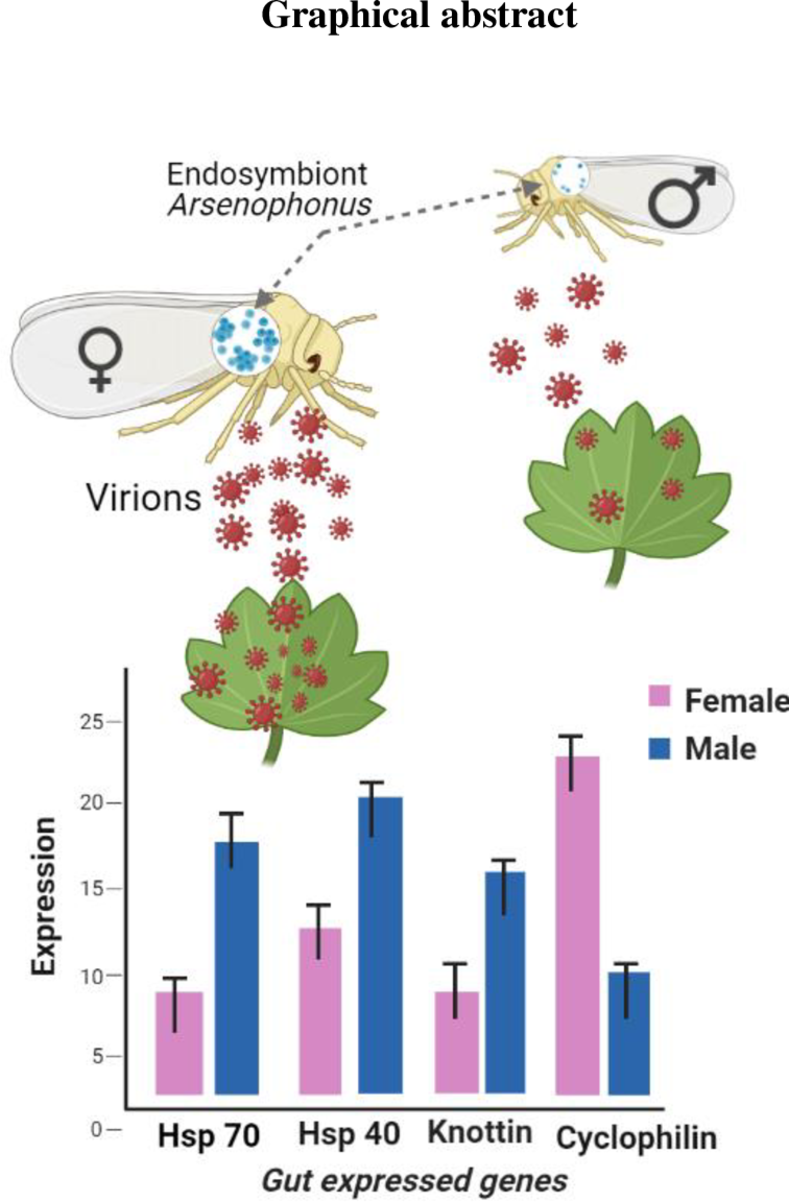

Supplement: S1 Graphical abstract — (TIF) [file pone.0259374.s004.tif]
