# Supplementary material for: Differential expression of gut protein genes and population density of Arsenophonus contributes to sex-biased transmission of Bemisia tabaci vectored Cotton leaf curl virus
Source: PLoS One. 2021 Nov 29;16(11):e0259374. doi: 10.1371/journal.pone.0259374 (PMC8629229; doi:10.1371/journal.pone.0259374)
Supplement: S3 Fig — (PDF) [file pone.0259374.s003.pdf]

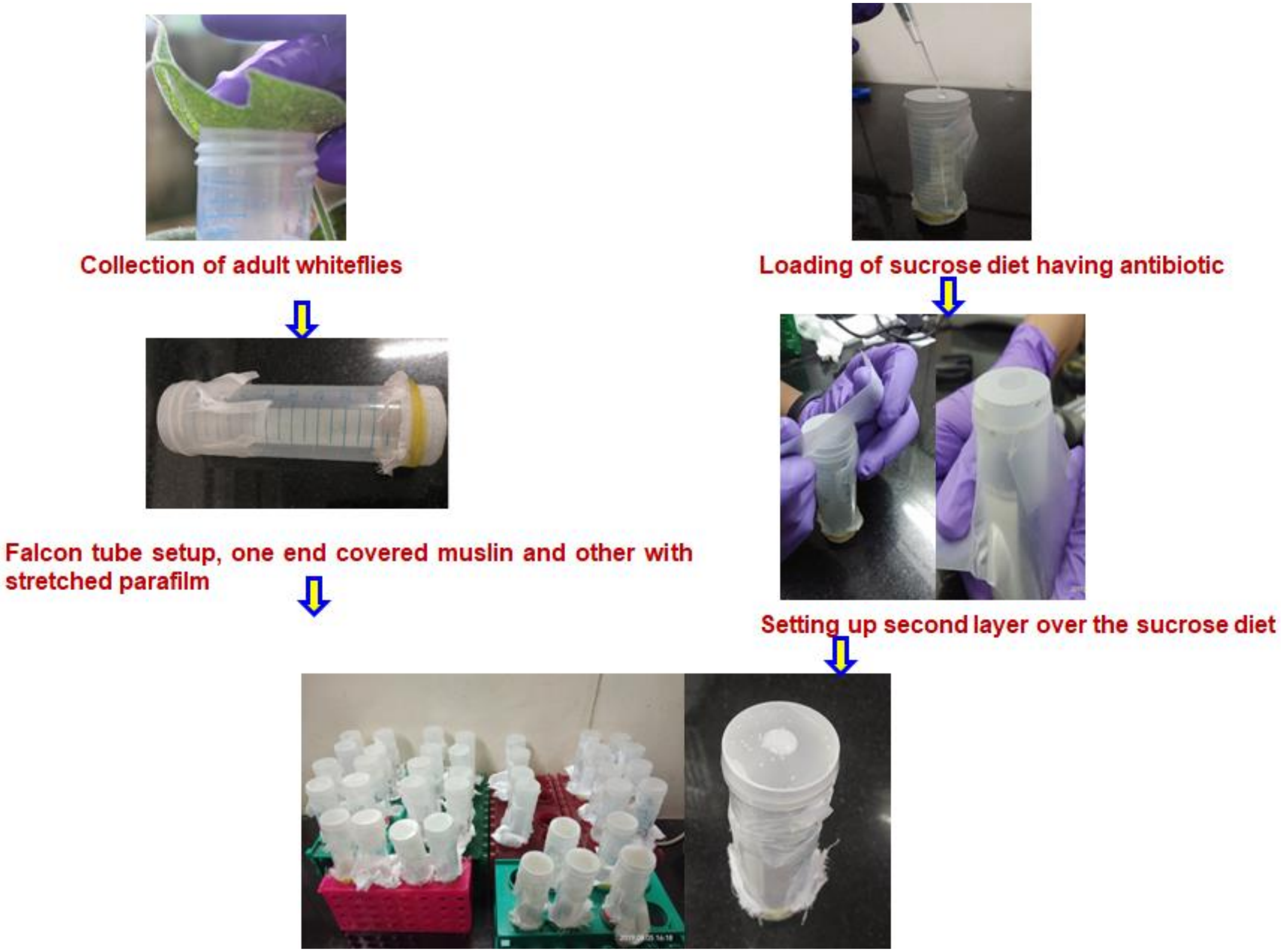
Collection of adult whiteflies
Falcon tube setup, one end covered muslin and other with stretched parafilm
Loading of sucrose diet having antibiotic
Setting up second layer over the sucrose diet
