# Supplementary material for: Differential expression of gut protein genes and population density of Arsenophonus contributes to sex-biased transmission of Bemisia tabaci vectored Cotton leaf curl virus
Source: PLoS One. 2021 Nov 29;16(11):e0259374. doi: 10.1371/journal.pone.0259374 (PMC8629229; doi:10.1371/journal.pone.0259374)
Supplement: Attachment — Submitted filename: Peer Review Report PLOS.docx [file pone.0259374.s005.docx]

This study by Singh et al focuses on the sex-biased transmission efficiency of Cotton Leaf Curl Virus (CLCuV) by *Bemisia tabaci*. The gender dependent differential variation was validated by using quantitative real-time PCR through the relative expression of CLCuV coat protein. The female whiteflies acquired and transmitted higher viral load compared to males. The authors identified that the higher density of *Arsenophonus* and differential expression of *Cyclophilin, Knottin*, *Hsp40*, *Hsp70* of the midgut genes may possibly contribute to higher vector competency to females compared to males. This study advances our understanding to key players involved in sex biased CLCuV transmission. Overall, the study is novel and well conceptualized, however there are some points which need to be addressed before consideration to publish.

Major comments:

1. The CLCuV coat protein real-time PCR primers used for this study should be tested on non-infected whitefly as well as non-infected plant to ensure there is not any non-specific binding.
2. Line 264-266 explained Fig 3c and indicating expression levels of CLCuV coat protein, but the figure legends are indicating these are *Arsenophonus* levels. Please update the figure legends.
3. As reported, there is differential level of expression of the genes in male and female so the efficiency of knock down (KD) using same amounts dsRNA for both male as well as female could be different and that may affect the observations. Indeed, the transmission efficiency of KD female is reduced more compared to KD males which is in consistent with the study. Ideally, it is important to normalize the gene expression in knockdown male as well as females. In short, authors should provide data for the expression levels of *Arsenophonus* in tetracycline fed/control male as well as female to validate the knock down for *Arsenophonus*. Also, it would have been interesting to have the comparative expression levels of GroEL during acquisition or transmission to strengthen and complement the data.
4. The authors should provide data for the expression levels of *Cyclophilin* in dsRNA fed/control male and female to validate the authenticity of knockdowns. Similar data should be provided for *Knottin*, *Hsp40*, *Hsp70* as proof of concept.
5. Fig.6c data plot should be in comparison to male versus GFP fed control. Similarly, for fig.6d data plot should be comparison to female versus GFP fed control. Please update the labels on data plots.
6. It was quite evident that the expression levels of *Arsenophonus,* *Knottin, Hsp40, Hsp70* in viruliferous flies are higher in comparison to non- viruliferous flies irrespective of gender. But the expression levels of *Cyclophilin* are lower in viruliferous flies in comparison to non-viruliferous flies. The authors should address that in the manuscript.

Minor comments:

1. The male and female should be represented with different color for better representation of the graphs.
2. The authors should label Y-axis for all the data plots for consistency throughout the manuscript.
3. The statistics analysis is missing for the graphs Fig.1 c, Fig.3 d, Fig.4 a, c, d, Fig.5 a, b, c, Fig.6 d, Fig.7 c.
4. Primers in Table 1 should be cross validated as the forward and reverse primers for HSP40 cannot be identical.
5. Please provide the details (accession number or Uniprot ID) in methods section for the CLCuV coat protein sequence used for designing the primers for real-time PCR. Also, adding details of the source of Cotton Leaf Curl Rajasthan Virus would be better.
6. It is also recommended to provide additional details in methods sections for the software or tools used to analyze real time data, statistical and image analysis.
7. Line 220 please provide the full form for DOI.
