# Supplementary material for: Differential expression of gut protein genes and population density of Arsenophonus contributes to sex-biased transmission of Bemisia tabaci vectored Cotton leaf curl virus
Source: PLoS One. 2021 Nov 29;16(11):e0259374. doi: 10.1371/journal.pone.0259374 (PMC8629229; doi:10.1371/journal.pone.0259374)
Supplement: Attachment — Submitted filename: Response to Reviewers.docx [file pone.0259374.s006.docx]

Dear Editor

We are grateful to the reviewers for spending their time for the critical and valuable comments in the manuscript. We have tried to address each and every query raised by the editor and reviewer. The point to point rebuttal has been given below:

| S# | Editors/ Reviewer’s comment | Author’s response |
| --- | --- | --- |
| **Editors comments:** | | |
| 1 | Please ensure that your manuscript meets PLOS ONE's style requirements, including those for file naming | Manuscript has been modified according to Plos one style as suggested. |
| 2 | We note that you have referenced (ie. Bewick et al. [5]) which has currently not yet been accepted for publication. Please remove this from your References and amend this to state in the body of your manuscript: (ie “Bewick et al. [Unpublished]”) as detailed online in our guide for authors | Reference has been deleted as per suggestion |
| 3 | We note that Figure 2 and Supplementary Figures 1, 2 and 3 in your submission contain copyrighted images. All PLOS content is published under the Creative Commons Attribution License (CC BY 4.0), which means that the manuscript, images, and Supporting Information files will be freely available online, and any third party is permitted to access, download, copy, distribute, and use these materials in any way, even commercially, with proper attribution.   We require you to either (1) present written permission from the copyright holder to publish these figures specifically under the CC BY 4.0 license, or (2) remove the figures from your submission: | Figure 2 and Supplementary Figures 1, 2 and 3 have been clicked by us in our lab/ greenhouse setup. So we do not need to seek any permissions for using these figures in our manuscript.  However, S1 Fig. contains pictures of cages purchased from Bugdrom Store (Taiwan). The permission from the manufacturer has been sought to use the picture of cages in manuscript (permission under the CC BY 4.0 license has been attached) |
| **Reviewer 1:** | | |
| 1 | The CLCuV coat protein real-time PCR primers used for this study should be tested on non-infected whitefly as well as non-infected plant to ensure there is not any non-specific binding. | The CLCuV coat protein primers has been validated on non-viruliferous and viruliferous whiteflies and plants. Details added in Fig 1. |
| 2 | Line 264-266 explained Fig 3c and indicating expression levels of CLCuV coat protein, but the figure legends are indicating these are *Arsenophonus* levels. Please update the figure legends. | Done |
| 3 | As reported, there is differential level of expression of the genes in male and female so the efficiency of knock down (KD) using same amounts dsRNA for both male as well as female could be different and that may affect the observations. Indeed, the transmission efficiency of KD female is reduced more compared to KD males which are in consistent with the study. Ideally, it is important to normalize the gene expression in knockdown male as well as females. In short, authors should provide data for the expression levels of *Arsenophonus* in tetracycline fed/control male as well as female to validate the knock down for *Arsenophonus*. Also, it would have been interesting to have the comparative expression levels of GroEL during acquisition or transmission to strengthen and complement the data. | We thanks reviewer for this comment. The knocked down after tetracycline feeding in female and male relative to control female and male whiteflies has been validated and added in main text along with figure and statistical data. As per suggestion comparative expression levels of GroEL during acquisition or transmission has been conducted and incorporated in Fig 4. |
| 4 | The authors should provide data for the expression levels of *Cyclophilin* in dsRNA fed/control male and female to validate the authenticity of knockdowns. Similar data should be provided for *Knottin*, *Hsp40*, *Hsp70* as proof of concept | The relative expression *Cyclophilin, Knottin*, *Hsp40* and *Hsp70* in dsRNA fed and control male and female has been validated using RT-PCR and statistical analysis was performed by Student t-Test. The data has been added in main text body and Fig 5, 6, 7 and 8 |
| 5 | Fig.6c data plot should be in comparison to male versus GFP fed control. Similarly, for fig.6d data plot should be comparison to female versus GFP fed control. Please update the labels on data plots. | Fig. 6c and 6d has been rephrased to updated to panel Fig. 7C and labels have been updated as per suggestions. |
| **Minor Comments** | | |
| 1 | The male and female should be represented with different color for better representation of the graphs. | Male and female has been represented by different colours in all the figures |
| 2 | The authors should label Y-axis for all the data plots for consistency throughout the manuscript. | Done |
| 3 | The statistics analysis is missing for the graphs Fig.1 c, Fig.3 d, Fig.4 a, c, d, Fig.5 a, b, c, Fig.6 d, Fig.7 c. | The data have been analysed by Student’s T test and this has been mentioned in the text |
| 4 | Primers in Table 1 should be cross validated as the forward and reverse primers for HSP40 cannot be identical. | This has been changed as suggested. |
| 5 | Please provide the details (accession number or Uniprot ID) in methods section for the CLCuV coat protein sequence used for designing the primers for real-time PCR. Also, adding details of the source of Cotton Leaf Curl Rajasthan Virus would be better. | Accessions have been mentioned in the text. Inadvertently we have written the virus name as Cotton Leaf Curl Rajasthan Virus in place of Cotton Leaf Curl Multan-Rajasthan Virus and this is the correct classification as per ICTV Code. The virus strain has been continuously maintained on cotton plants and is crosschecked time to time for its purity. |
| 6 | It is also recommended to provide additional details in methods sections for the software or tools used to analyze real time data, statistical and image analysis. | Details have been added in materials methods. |
| 7 | Line 220 please provide the full form for DOI. | The DOI has been replaced by DPI (Days Post Inoculation) |
| **Reviewer 2** | | |
| 1 | Whitefly, Bemisia tabaci (Gennadius) is an important pest of cotton as vector of Cotton Leaf Curl Virus (CLCuV) and the paper showed B. tabaci AsiaII-1 haplotype showing higher virus transmission efficiency of females than males. The transmission is related to the density of endosymbiont Arsenophonus, but the expression of some midgut proteins genes i.e. Cyclophilin, Knottin, Hsp40, Hsp70 may be possibly associated with the vector competency of both sex. All the results might not give us explicit conclusions that females treated by knocked-down or antibiotics could directly show higher competency to transmit the virus.  My suggestion is that authors just focus on the role of one of endosymbionts and its midgut protein on the sex-biased transmission of whitefly. However, the title “Is Cotton Leaf Curl Virus transmission by Bemisia tabaci (Gennadius) (Hemiptera: Aleyrodidae) sex-biased?” does not fully explain and cover all main ideas of the manuscript, i.e. the relation the sex-biased of transmission with endosymbionts and their proteins. | This is a good suggestion and keeping in view the reviewers comments we have revised the title of manuscript title highlighting Arsenophonus and gut proteins. |
