# Supplementary material for: Differential expression of gut protein genes and population density of Arsenophonus contributes to sex-biased transmission of Bemisia tabaci vectored Cotton leaf curl virus
Source: PLoS One. 2021 Nov 29;16(11):e0259374. doi: 10.1371/journal.pone.0259374 (PMC8629229; doi:10.1371/journal.pone.0259374)
Supplement: Attachment — Submitted filename: review comments_.docx [file pone.0259374.s007.docx]

This study by Singh et al, provides substantial evidence of correlation between sex based differential gene expression and transmission efficiency of Cotton Leaf Curl Virus (CLCuV) by Bemisia tabaci. The study was conducted using B. tabaci Asia II-1 halotype and the virus transmission efficiency of females was reported significantly higher over males. The authors have addressed majority of the comments from both the reviewers. However, I have mentioned few important concerns which should be addressed before consideration for publishing. Therefore, I would recommend acceptance with minor revision.

1- The color codes for the data plots are not consistent for example female are orange and controls are white in Fig-1 but in Fig 3B and 3C both male and female are blue, and controls became orange. Please try to maintain consistent colors throughout the manuscript.
2- Please provide the accession number if available for the coat protein sequence of the CLCuMuV-Ra which was used to design the real time primers for this study.
3- The real time PCR reaction conditions mentioned in lines 119-121 are different from lines 150-152, but both were used for the detection of **coat protein** either from whiteflies or from infected plants. Please justify.
4- Lines 229 and 230 please correct nl to microliters.
5- Fig1 E the fluid loss assay data is not clear as blue dots are not easy to visualize.
6- Lines 279-280 belong to Fig 3 legends to my understanding. Please correct accordingly.
7- The relative levels of Arsenophonus in non-viruliferous whiteflies are significantly higher than viruliferous whiteflies (Fig 3A) which is not corelating with the relative levels of GroEL. The relative amounts of GroEL are lower in non-viruliferous whiteflies in comparison with viruliferous whiteflies (Fig 4A) Please cross-check if there is labelling error in data plots of Fig3A or justify.
8- Line 343 the expression levels of cyclophilin in non-viruliferous males and females **are not** at par in Fig 5A. As Fig 5A data plots clearly indicate that females have higher levels of cyclophilin in viruliferous as well as non-viruliferous whiteflies. Please modify accordingly.
9- Fig 7B the control females have higher levels of Hsp40 than control males while Fig 7A clearly states females express lesser amounts of Hsp40. Similarly, Fig 8B the control females have slightly higher levels of Hsp70 than control males which is contradictory to data in 8A. Please correct the controls accordingly to support the data for 7A and 8A.
10- Fig 5C, 6C, 7C, 8C the control females have lower virus transmission rate than control males while Fig 1 clearly indicates female have higher transmission rate than males it is not consistent with the hypothesis throughout the manuscript. Please add corrections accordingly or provide justification.
